# Rapid Population Growth throughout Asia’s Earthquake-Prone Areas: A Multiscale Analysis

**DOI:** 10.3390/ijerph15091893

**Published:** 2018-08-31

**Authors:** Yinyin Dou, Qingxu Huang, Chunyang He, Shiting Meng, Qiang Zhang

**Affiliations:** 1 Center for Human-Environment System Sustainability (CHESS), State Key Laboratory of Earth Surface Processes and Resource Ecology (ESPRE), Beijing Normal University, Beijing 100875, China; douyinyin1986@163.com (Y.D.); qxhuang@bnu.edu.cn (Q.H.); gloria_meng@126.com (S.M.); 2 Academy of Disaster Reduction and Emergency Management, Faculty of Geographical Science, Beijing Normal University, Beijing 100875, China; zhangq68@bnu.edu.cn; 3 School of Natural Resources, Faculty of Geographical Science, Beijing Normal University, Beijing 100875, China; 4 Key Laboratory of Environmental Changes and Natural Disaster, Ministry of Education, Beijing Normal University, Beijing 100875, China; 5 State Key Laboratory of Earth Surface Processes and Resource Ecology (ESPRE), Beijing Normal University, Beijing 100875, China

**Keywords:** most seismically hazardous area, vulnerable population, earthquake exposure, urban population, Asia

## Abstract

Assessing the changes of the population living throughout the most seismically hazardous area (MSHA) constitutes an important foundation for seismic risk assessment. However, the changes of the population living in the MSHA of Asia, which exhibits the highest number of earthquake related fatalities, were poorly understood. Therefore, this study analyzed the changes of the population in the MSHA between 2000 and 2015 at the continental, subcontinental, and national scales. We found that the population, especially the vulnerable population (i.e., children under or equal to the age of 14 and elderly people over or equal to the age of 65), in Asia’s MSHA increased rapidly between 2000 and 2015. The population in the MSHA increased by 185.88 million with a growth rate of 20.93%, which was 3.38% greater than that in the non-MSHA region. Meanwhile, the vulnerable population in the MSHA increased by 63.65 million with a growth rate of 19.73%. The increase of the vulnerable population in the MSHA was 19.93% greater than that in the non-MSHA region. We also found that urban population growth was a major factor impacting the increase in both the population and the vulnerable population throughout Asia’s MSHA. Therefore, attention should be paid to the changes of the population in Asia’s MSHA, whilst it is imperative to execute strict building codes and select the development location more carefully in the MSHA.

## 1. Introduction

The most seismically hazardous area (MSHA) represents a region where earthquakes that could cause casualties and property damage may occur [1,2,3]. The MSHA is a region where earthquake destruction and related losses concentrate [2,4]. It constitutes the main region of seismic risk concern [3,5,6,7]. The population living in the MSHA is a vital component of earthquake exposure, and changes of it can lead to the changes in seismic risk [1,2,3,8,9]. Among the people living in the MSHA, children and elderly people are more susceptible than others to an earthquake disaster and require extra earthquake disaster risk management measures to have the same level of risk as others [10]. Therefore, the children and the elderly people in the MSHA can be defined as vulnerable people as they are less physically capable to avoid earthquake disasters and/or recover from earthquake disasters [11,12].

Asia is the most populous continent in the world. In 2015, the total population of Asia accounted for nearly 60% of the global population [13]. Meanwhile, Asia is also the continent with the highest earthquake related fatalities [14,15]. Between 1970 and 2008, 84% of global earthquake related fatalities were reported in Asia [14]. Recently, Asia has been undergoing rapid urbanization. The percentage of the population living in urban areas increased at an annual average rate of 0.71% from 2000 to 2015, which was the highest in the world [13]. The amount, demographic structure, and spatial distribution of the population living in Asia’s MSHA have altered substantially in the context of urbanization [16]. A previous study found that the population of Asia living in areas with an earthquake intensity of greater than or equal to VII (under the Modified Mercali Intensity of VII to X, the shaking intensity varies from very strong to extreme and the damage to built structures ranges from considerable to completely destroyed [17]) increased by 44.09% from 1990 to 2015 [18]. Therefore, a timely and accurate assessment of the changes of the population throughout Asia’s MSHA has become a critical scientific issue for seismic risk prevention.

Several researchers have estimated the status and changes of the population within Asia’s MSHA at multiple scales. In terms of the population distribution, Nojima et al. [19] found that 80% of the county-level population in Japan was living in areas characterized by high seismic hazards in 2005. In terms of population changes, He et al. [20] found that the population in China’s MSHA increased by 33.63% from 1990 to 2010, which was twice the average growth rate of the population of China. Freire et al. [16] found that there was an increasing population trend in seismically hazardous areas across 11 megacities in Asia from 1950 to 2010.

However, the changes in the population, particularly with regard to the vulnerable people living in Asia’s MSHA, remained poorly understood. The research gap is primarily due to the incomparability of the population data among different countries and the lack of age structure information in those population data [21,22,23,24]. Thus, it was impossible to analyze the changes of the vulnerable population living in Asia’s MSHA. The recently published WorldPop datasets have provided an important data source for studying the changes of the population in Asia’s MSHA [25]. Covering the entire continent of Asia, the WorldPop version 2 dataset published in 2017 provides estimates of the population from 2000 to 2020 with a spatial resolution of 1 km [26]. The accuracies of the spatial population distributions in the WorldPop version 2 dataset were significantly improved since the version 1 dataset was produced in 2011 using the downscaling method, which aggregates each element in the population data within a 100-m spatial resolution [27]. Moreover, compared with other freely available population data, the WorldPop datasets include, not only total population data, but also demographic data for different ages, including elderly people, adults, and children [26]. The new dataset has been successfully applied to relevant research at the continental and national scales during recent years [26,28,29].

The objective of this study was to analyze the changes of the population living in Asia’s MSHA from 2000 to 2015. To achieve this goal, we first analyzed the changes in the total population from 2000 to 2015 living in the MSHA at continental, subcontinental, and national scales. Then, we analyzed the changes in the vulnerable population during the same period living in the MSHA at the abovementioned three scales. Finally, we discussed the reliability, causes, consequences, and implications of the results. Such an investigation plays an essential role in understanding the population changes in Asia’s MSHA and evaluating earthquake disaster losses.

## 2. Materials and Methods 

### 2.1. Study Area

**Figure 1:**
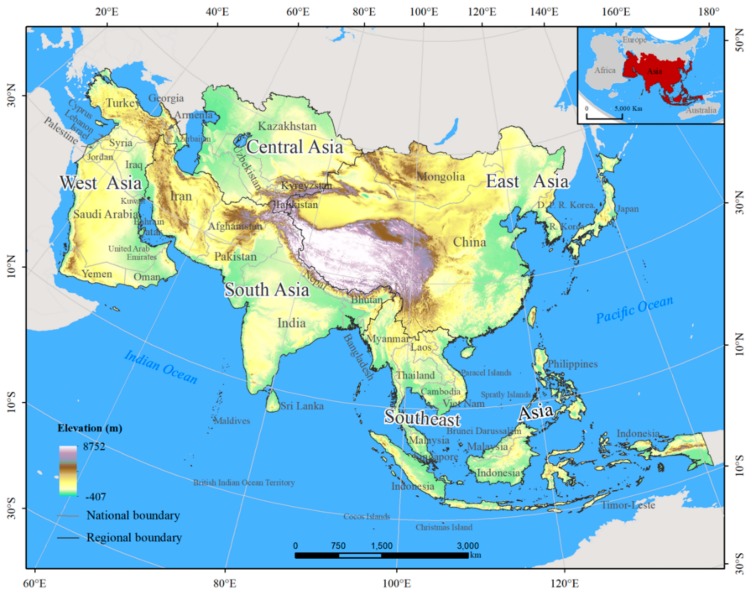
The study area.

The study area, which extends from 145°48′ W to 25°41′ E and from 55°27′ N to 11°7′ S, is composed of the continent of Asia and consists of East Asia, Central Asia, South Asia, West Asia, and Southeast Asia (Figure 1). The subcontinental and national boundaries are from the Data Center for Resources and Environmental Sciences, Chinese Academy of Sciences (http://www.resdc.cn) [30]. At the national scale, we focused on 53 countries and regions throughout Asia, including five countries in East Asia (e.g., China, Japan, and Mongolia), five countries in Central Asia (e.g., Kazakhstan, Turkmenistan, and Kyrgyzstan), 10 countries and regions in South Asia (e.g., Afghanistan, Nepal, and Iran), 18 countries in West Asia (e.g., Turkey, the United Arab Emirates, and Azerbaijan), and 15 countries and regions in Southeast Asia (e.g., Indonesia, the Philippines, and Timor-Leste). Because Russia stretches over the European and Asian continents, it was not included in the study area [30]. The terrain is dominated by plateaus and mountains, and the central part is higher than the surrounding areas [31]. The continent of Asia is primarily located among the Pacific plate, the Eurasian plate, and the Indian plate and exhibits some of the most frequent and strong earthquakes worldwide [32].

### 2.2. Data

In this study, we used five types of data. First, the population data of Asia between 2000 and 2015 were obtained from the WorldPop version 2 dataset published by the GeoData Institute of the University of Southampton in 2017. The data have a spatial resolution of 1 km (http://www.worldpop.org.uk/data/) and consist of both the total population and the population in each 5-year age group in Asia.

The second type of data comprises peak ground acceleration (PGA) data for Asia that were published in 1999 by the Global Seismic Hazard Assessment Program with the support of the International Council of Scientific Unions (ICSU) (http://www.seismo.ethz.ch/static/gshap). The PGA data are depicted with a 10% chance of exceedance in a 50-year period corresponding to a return period of 475 years. The spatial resolution of the PGA data is 0.1 degrees [33]. The PGA data were compiled using a uniform procedure based on historical earthquake data throughout Asia following the cooperation of earthquake experts among Asian countries for many years. The resulting map eliminates the boundary discrepancies in seismic hazard maps among different Asian countries, and it is the only seismic hazard map of Asia with a full coverage produced using a consistent method [34].

The third dataset comprises socioeconomic data between 2000 and 2015 for Asia that were gathered from the World Bank (http://data.worldbank.org/). These socioeconomic data include a number of socioeconomic indicators, including the population density, urban population, birth rate, infant mortality rate, gross domestic product (GDP), and GDP per capita, for Asian countries.

The fourth type of data is urban land data for Asia in 2000 and 2015 from the HYDE dataset, which was published by the PBL Netherlands Environmental Assessment Agency (ftp://ftp.pbl.nl/hyde) [35]. The new version of the HYDE dataset (version 3.2) (PBL Netherlands Environmental Assessment Agency, Bezuidenhoudseweg 30, the Netherlands), which includes land use data, was published in 2016 [36]. The period of coverage is 10 000 BCE to 2015 CE. Urban land data is also provided, and the spatial resolution of the data is 0.083 degrees. After collecting all of the data, we resampled these data on an Albers projection to a spatial resolution of 1 km.

### 2.3. Methods

#### 2.3.1. Determining the MSHA

**Figure 2:**
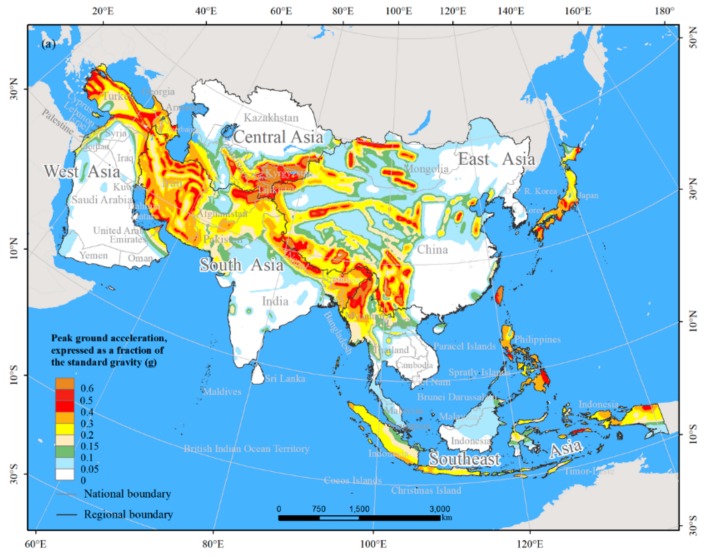

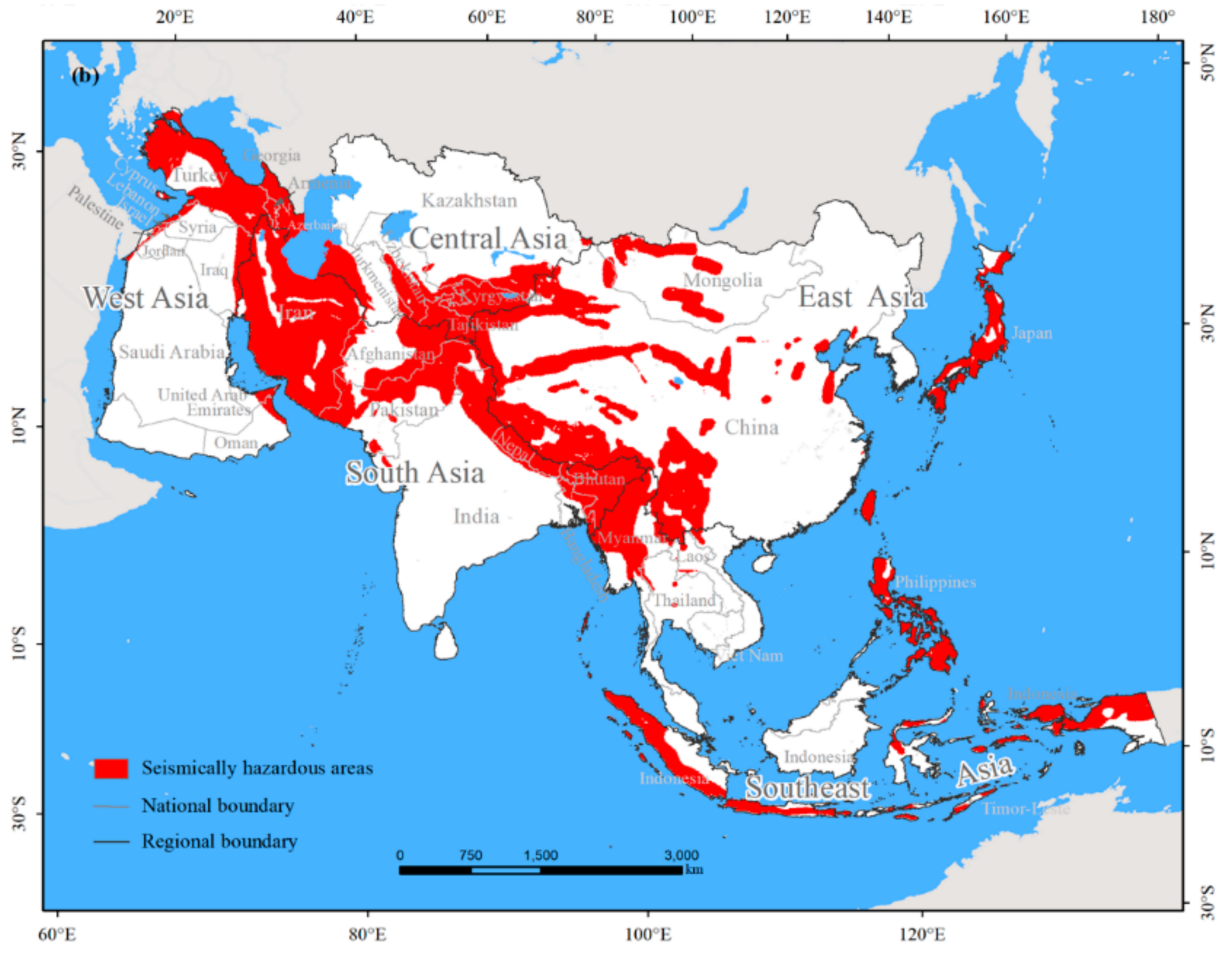
Seismic PGA map. (**a**) The PGA with a 10% probability of exceedance in 50 years; (**b**) The most seismically hazardous area (MSHA) with PGA values greater than or equal to 0.2 g.

At present, there are mainly two methods for determining the MSHA [2,19,37,38]. One method was based on the seismic intensity. For example, Nojima et al. [19] regarded the MSHA as the areas with a 3% probability of exceeding seismic intensity 6 lower for 30 years defined by Japan’s Meteorological Agency. Jaiswal et al. [38] defined that the MSHA were the areas with the Modified Mercalli Intensity greater than or equal to VII. However, the extent of the MSHA determined by the seismic intensity are not comparable among countries, since different countries adopted different measures for seismic intensity. The other method was based on the PGA value. This method was widely used for determining the MSHA [2,20,39], because it eliminated the discrepancies in seismic intensity among different countries. Therefore, in this study, the spatial distribution of the MSHA was determined using the PGA data. According to the criterion used by Holzer and Savage [2], the MSHA was defined as the areas with PGA values greater than or equal to 0.2 g (Figure 2). The MSHA corresponds to the areas with a value of VII on the instrumental seismic intensity scale according to the United States Geological Survey [17].

#### 2.3.2. Analyzing the Changes in the Total Population in the MSHA

Based on the spatial distributions of the MSHA and the population data, we calculated the changes in the total population (*Pop_(∆k, MSHA)_*) living in the MSHA between 2000 and 2015 using the below equation,

*Pop_(∆k, MSHA)_* = (Ʃ_i=1_^n^*pop_(k2, i)_* − Ʃ_i=1_^n^*pop_(k1, i)_*) × *D_(MSHA, i)_* (1)

where *Pop_(∆k, MSHA)_* denotes the changes in the total population living in the MSHA from the year *k*_1_ to the year *k*_2_; *pop_(k_*_1*, i)*_ and *pop_(k_*_2*, i)*_ refer to the total population in the *i*th pixel in the year *k*_1_ and *k*_2_, respectively; and *D_(MSHA, i)_* represents the class value, which is set to 1 if the *i*th pixel is located in the MSHA and is otherwise set to 0.

#### 2.3.3. Analyzing the Changes in the Vulnerable Population in the MSHA

The vulnerable population refers to persons or groups whose characteristics and situations will affect their capacities to anticipate, cope with, resist, and recover from the impact of a natural hazard [10,39]. Because children and elderly persons are prone to injury or death during an earthquake, they are usually regarded as the vulnerable population [40,41,42]. In this paper, persons aged between 0 and 14 were regarded as children, and persons aged 65 and above were regarded as the elderly population [43]. The equations used to calculate the changes in the vulnerable population in the MSHA (*Vul_(∆k, MSHA)_*) are as follows,

*Vul*_(∆*k*, *MSHA*)_ = *Chil*_(∆*k*, *MSHA*)_ + *Eld*_(∆*k*, *MSHA*)_ (2)

of which,

*Chil*_(∆*k*, *MSHA*)_ = (Ʃ_i=1_^n^*Chil*_(*k*2, *i*)_ − Ʃ_i=1_^n^*Chil*_(*k*1, *i*)_) × *D*_(*MSHA,i*)_ (3)

*Eld*_(∆*k*, *MSHA*)_ = (Ʃ_i=1_^n^*Eld*_(*k*2, *i*)_ − Ʃ_i=1_^n^*Eld*_(*k*1, *i*)_) × *D*_(*MSHA,i*)_ (4)

where *Chil_(∆k, MSHA)_* and *Eld_(∆k, MSHA)_* refer to the changes in the child population and elderly population, respectively, living in the MSHA from the year *k*_1_ to the year *k*_2_; *Chil_(k_*_1*, i)*_ and *Chil_(k_*_2*, i)*_ are the child populations in the *i*th pixel in the years *k*_1_ and *k*_2_, respectively; and *Eld_(k_*_1*, i)*_ and *Eldl_(k_*_2*, i)*_ are the elderly populations in the *i*th pixel in the years *k*_1_ and *k*_2_, respectively.

Then, we analyzed the changes in the total population in the MSHA at the continental, subcontinental, and national scales. At the subcontinental scale, the total population changes in the MSHA of East Asia, Central Asia, South Asia, West Asia, and Southeast Asia were analyzed. At the national scale, the national administrative boundaries were overlapped with the boundaries of the MSHA. After this overlap, the MSHA was detected within 36 countries throughout Asia. Then, we analyzed the changes in the total population in the MSHA across these 36 countries. In addition, we further evaluated the changes in the vulnerable population in the MSHA at the abovementioned three scales.

## 3. Results

### 3.1. Features of the MSHA in Asia

The total area of the MSHA in Asia was 8.99 million km^2^, which accounts for 28.85% of Asia’s total land area (Figure 2b, Table 1). The MSHA was concentrated in South and East Asia. The MSHA in South Asia was the largest at the subcontinental scale with a total area of 3.04 million km^2^, followed by the area of East Asia MSHA of 2.72 million km^2^. The total area of the MSHA in these two regions accounted for approximately 60% of the total area of the MSHA in Asia. The areas of the MSHA in Southeast Asia, West Asia, and Central Asia were 1.38 million, 9.64 million, and 0.88 million km^2^, accounting for 15.31%, 10.72%, and 9.84% of the total area of the MSHA in Asia, respectively (Table 1).

At the national scale, the areas of the MSHA in China, Iran, Indonesia, Turkey, and India individually exceeded 50 km^2^ (Figure 2b). The areas of the MSHA among those five countries collectively accounted for 60.40% of the total area of the MSHA in Asia. Among them, the area of the MSHA in China was the largest and reached up to 2.15 million km^2^, which accounted for 23.93% of the total area of the MSHA in Asia. The areas of the MSHA in Iran, Indonesia, Turkey, and India were 1.44 million, 0.71 million, 0.60 million, and 0.53 million km^2^, accounting for 16.02%, 7.92%, 6.62%, and 5.92% of the total area of the MSHA in Asia, respectively (Table A1).

### 3.2. Population Changes among MSHA between 2000 and 2015

**Figure 3:**
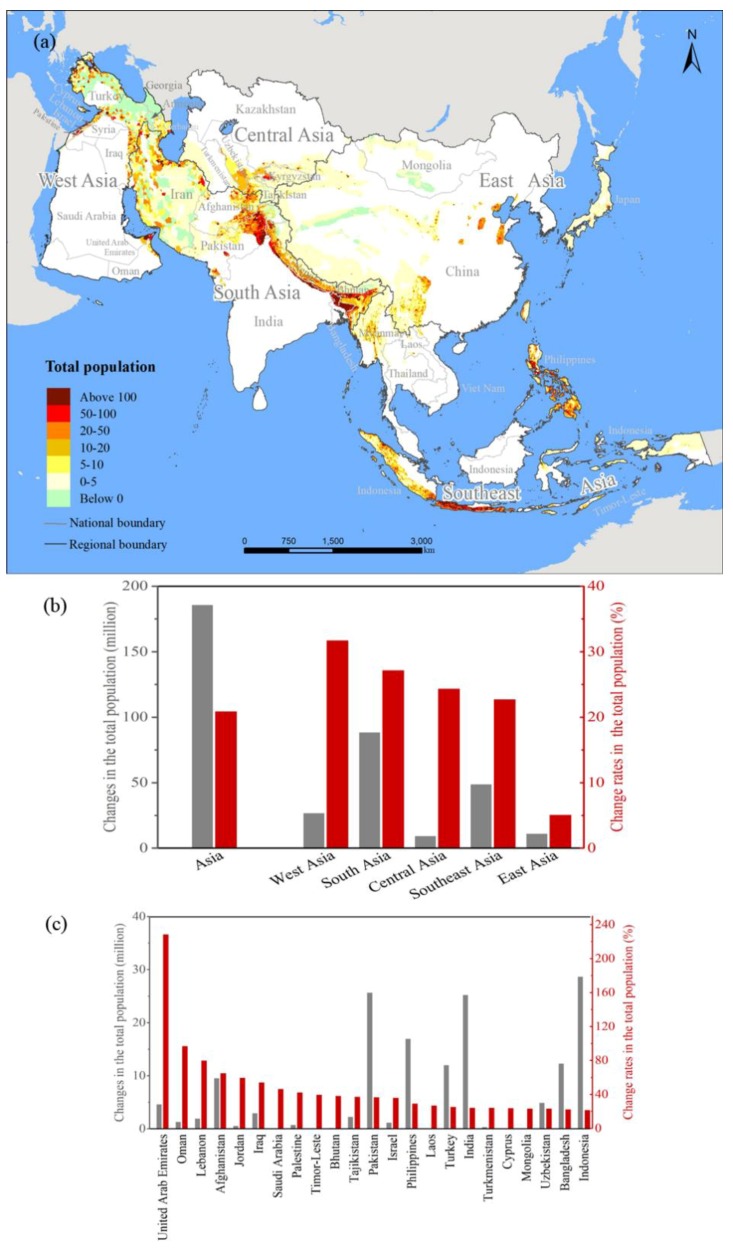
Changes in the total populations living among different MSHA from 2000 to 2015. (**a**) The total population density, unit: people/km^2^; (**b**) The change rates in the total population in the MSHA of Asia; (**c**) The change rates in the total populations living in the MSHA of the selected countries. (The countries were selected when the total population in an MSHA increased from 2000 to 2015).

The population living in Asia’s MSHA increased rapidly from 0.88 billion people in 2000 to 1.07 billion people in 2015 with a growth rate of 20.93% (Figure 3a, Table 2). During that same period, the total population living in the non-MSHA region of Asia increased by 17.55%, from 2.80 billion people to 3.29 billion people. In other words, the increase of the former was 3.38% higher than that of the latter.

At the subcontinental scale, the increase of the population living in the MSHA of West Asia was the highest (Figure 3b, Table 2). The population in the MSHA of West Asia increased by 31.78%, from 85.16 million people in 2000 to 112.22 million people in 2015. That is, the increase of the population in the MSHA of West Asia was 1.52 times the average growth rate among the MSHA of Asia. However, the increase of the population in the MSHA of East Asia was the smallest (5.15%), as the population therein increased from 222.06 million people in 2000 to 233.47 million people in 2015 (Table 2). During that same period, the populations within the MSHA of South Asia, Central Asia and Southeast Asia increased by 27.21%, 24.41% and 22.78%, which was 88.73 million, 9.57 million and 49.11 million people, respectively (Figure 3b, Table 2).

At the national scale, the growth rates of the populations living within the MSHA of 23 countries were larger than the average growth rate of the population in the MSHA of Asia (i.e., 20.93%). Among those 23 countries, the increase of the population in the MSHA of the United Arab Emirates was the highest (Figure 3c, Table A2), as the population living therein increased from 2.03 million people in 2000 to 6.68 million people in 2015 with a growth rate of 228.41%, which was 10.91 times the average growth rate of the population in Asia’s MSHA during that period. The increase of the populations in the MSHA of seven countries, including Oman, Lebanon, and Afghanistan, ranged from 42.39% to 97.24% (Figure 3c, Table A2). The increase of the populations in the MSHA of 15 countries, including East Timor, Bhutan, and Tajikistan, fluctuated between 21.68% and 39.70%. Meanwhile, the increase of the populations living within the MSHA of 11 countries, including Azerbaijan, Nepal, and Kyrgyzstan, were lower than the average growth rate of the population in Asia’s MSHA and varied between 0.64% and 20.27% (Figure 3c, Table A2).

### 3.3. Vulnerable Population Changes among MSHA between 2000 and 2015

**Figure 4:**
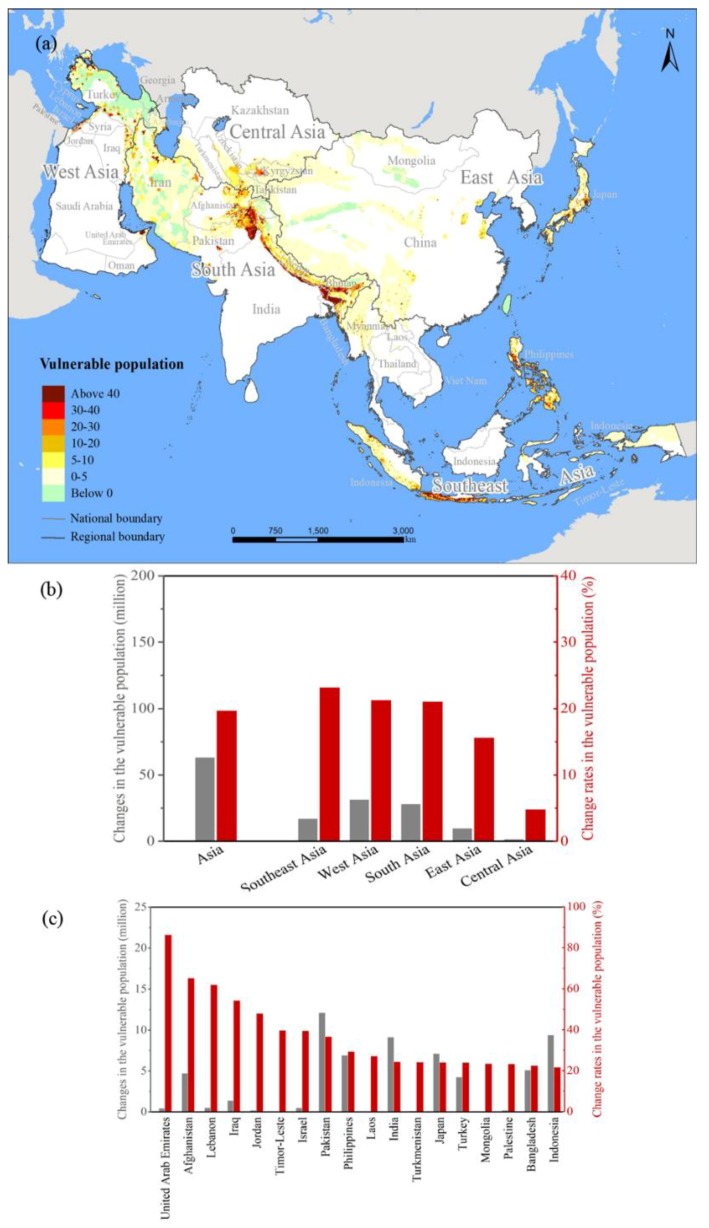
Changes in the vulnerable populations living among different MSHA from 2000 to 2015. (**a**) The vulnerable population density, unit: person/km^2^; (**b**) change rates in the vulnerable population in the MSHA of Asia; and (**c**) change rates in the vulnerable populations in the MSHA of the selected countries.

The vulnerable population living in Asia’s MSHA also increased rapidly from 321.94 million people in 2000 to 385.47 million people in 2015 with a growth rate of 19.73% (Figure 4a, Table 3). Meanwhile, the vulnerable population living in the non-MSHA region of Asia decreased from 1013.47 million people to 1011.40 million people with a growth rate of −0.20%. In other words, the growth rate of the former was 19.93% greater than that of the latter.

At the subcontinental scale, the increase of the vulnerable population living in the MSHA of Southeast Asia was the highest (Figure 4a, Table 3), as the vulnerable populace therein increased from 75.04 million people in 2000 to 92.47 million people in 2015 with a growth rate of 23.23%. Such a growth rate was 1.18 times the average growth rate of the vulnerable population in the MSHA of Asia during that period. However, the increase of the vulnerable population in the MSHA of Central Asia was the smallest, as the vulnerable population living therein increased from 15.92 million people in 2000 to 16.69 million people in 2015, during which period the corresponding increase reached only 4.84%. During that same period, the increase of the vulnerable populations living in the MSHA of West Asia, South Asia, and East Asia were 21.33%, 21.11%, and 15.65%, respectively, and the number of vulnerable people in each of those three regions increased by 6.93 million, 28.37 million, and 10.03 million people, respectively (Table 3).

At the national scale, the growth rates of the vulnerable populations living among the MSHA of 18 countries were larger than the average growth rate of the vulnerable population in Asia’s MSHA, which was 19.73% (Figure 4c, Table A3). Among those 18 countries, the increase of the vulnerable population in the MSHA of the United Arab Emirates was the highest (Figure 4c, Table A3), as the vulnerable population therein increased from 0.54 million people in 2000 to 1.01 million people in 2015 with a growth rate of 86.33% (Figure 4c, Table A3), which was 4.37 times the average growth rate of the vulnerable population in Asia’s MSHA during that period. The increase of the vulnerable populations in the MSHA of four countries, i.e., Afghanistan, Lebanon, Iraq, and Jordan, ranged from 47.91% to 65.16% (Figure 4c, Table A3). The increase of the vulnerable populations in the MSHA of 13 countries, e.g., Israel, Pakistan, and East Timor, fluctuated between 21.68% and 39.70%. Meanwhile, the increase of the vulnerable populations living within the MSHA of 12 countries, e.g., Kazakhstan, Vietnam, and Oman, were smaller than that of the vulnerable population in Asia’s MSHA. The vulnerable populations in the MSHA of those 12 countries grew by 1.70 thousand to 2.87 million people with growth rates between 2.40% and 18.19% (Figure 4c, Table A3).

## 4. Discussion

### 4.1. Utilizing WorldPop Datasets Allows for an Effective Analysis of the Changes of the Population in the MSHA

Adopting the WorldPop datasets to assess the changes of the population living in Asia’s MSHA has three advantages. First, the population information in the WorldPop datasets is comparable among different countries. The WorldPop datasets are acquired using a uniform method [28] to provide population distribution information across both regional and national scales [26]. In contrast, the population data in statistical datasets are less comparable among different countries, because the statistical objects of populations vary among those countries [44].

Second, the WorldPop datasets have a high level of accuracy. The WorldPop datasets, which have a spatial resolution of 0.0083 degrees (approximately 1 km), were obtained based on the downscaling method, which aggregates population data elements within a spatial resolution of 100 m, thereby improving the spatial resolution and accuracy of the population distribution data significantly [26]. However, the other freely available population datasets that cover all of Asia, including the population data with a 10 km spatial resolution from HYDE [21,36] and the population data with a spatial resolution of 5 km from the GPW database, the GRUMP database, and the GRID database [22,23], have relatively lower spatial resolutions. Therefore, these datasets cannot accurately depict the spatial distribution of the population in Asia’s MSHA.

Third, the WorldPop datasets can be used to analyze the changes of the vulnerable population in the MSHA. The WorldPop datasets include demographic data of different age groups in Asia that can be used to evaluate the changes of the vulnerable population. However, the other above-mentioned spatial population datasets do not provide this type of information [20,21,22,23,45], and consequently, they do not possess the ability to be used to analyze the changes of the vulnerable population in the MSHA.

Meanwhile, the results of our study based on the WorldPop datasets are in accordance with those of previous studies. For example, Djordjević et al. [18] found that the population living in Asia among the zones with probable maximum earthquake intensities of greater than or equal to VII increased rapidly by 23.37% between 2000 and 2015. He et al. [20] found that the population in the MSHA of China also increased by 32.52 million people between 1990 and 2010 with a rapid growth rate of 33.63%. Freire et al. [16] also found that the population living in seismically hazardous areas across the 11 megacities of Asia increased between 1950 and 2010. Therefore, based on the WorldPop datasets, the changes of the population in Asia’s MSHA over the past 15 years could be analyzed effectively and accurately.

### 4.2. Urban Population Growth Was a Major Factor Impacting the Increase in Both the Population and the Vulnerable Population in Asia’s MSHA

Following the methods by Tao et al. [46] and Chatterjee et al. [47], we further analyzed the relationship between the socioeconomic development and the population growth throughout Asia’s MSHA using correlation analysis and multiple general linear model (GLM) regression techniques to explore the factors associated with the rapid population growth in Asia’s MSHA. With reference to Ma et al. [48], the two methods are complementary to each other when used properly together. Specifically, the Pearson correlation can be used to scope potentially important factors and seek the best predictive models. While the stepwise multiple linear regression can be adopted to solve the problem of multicollinearity between different factors and further identify the key factors. Based on the accessibility of the data, we selected seven national-scale socioeconomic factors, including the population density, urban population, birth rate, mortality rate, urban land area, GDP, and GDP per capita.

We found that the urban population growth played a leading role in the rapid growth of the population in Asia’s MSHA over the past 15 years. The population growth in Asia’s MSHA between 2000 and 2010 was significantly correlated with the population density growth, urban population growth, and urban land area growth in Asia, and the Pearson correlation coefficients for the three factors were each larger than 0.87 (*p* < 0.001) (Table 4). Among those three factors, the population density growth had the largest correlation coefficient (*r* = 0.99, *p* < 0.001) (Table 4). The GLM regression results further indicated that the urban population growth accounted for 65.87% of the variation in the population living in the MSHA. Meanwhile, the population density growth, urban land area growth, and GDP growth can account for the additional 32.82%, 0.37%, and 0.06%, respectively, of the variation in the population in the MSHA (Table 4).

In addition, we found that the urban population growth also played a leading role in the rapid growth of the vulnerable population in Asia’s MSHA during the same period. The vulnerable population growth in Asia’s MSHA between 2000 and 2015 exhibited significant and positive correlations with the population density growth, urban population growth, and urban land area growth in Asia. The Pearson correlation coefficients for the three factors were each larger than 0.57 at the significance level of 0.001 (Table 4). The GLM regression results also suggested that the urban population growth accounted for 50.95% of the variation in the vulnerable population in the MSHA. The changes in the population density, urban land area, mortality rate, and GDP accounted for the additional 26.19%, 1.18%, 0.56%, and 0.11% in the variations, respectively (Table 4).

In the context of rapid urbanization in Asia, seismic risk prevention measures should be fully considered in future land use planning [49]. We suggest that Asian countries with massive and rapid population growth in the MSHA (e.g., Pakistan, Iraq, and Afghanistan, Table A2) should strengthen their building codes. Furthermore, the countries in Asia exhibited large and rapid increases in their vulnerable MSHA populations (e.g., Pakistan, Iraq, and Afghanistan, Table A3) should improve the earthquake-resistance capacity of schools and hospitals for the children and elderly people, and also equip the vulnerable population with the knowledge of seismic disaster prevention and mitigation.

### 4.3. More Attention Should Be Paid to Demographic Changes in the MSHA

Historical data indicated that Asia’s MSHA broadly exhibited high-frequency, high-intensity, and high-fatality earthquakes. More than 80% of the 1824 earthquakes reported throughout Asia between 1900 and 2009 with magnitudes of greater than or equal to 5.5 occurred in Asia’s MSHA (Table A4). In addition, 84.08% of the earthquakes with magnitudes of greater than or equal to seven occurred in Asia’s MSHA (Table A4). Among these earthquakes, five of the six earthquakes with death tolls larger than 50,000 people, including the 1920 Haiyuan earthquake in China, the 1948 Ashgabat earthquake in Turkmenistan, the 1976 Tangshan earthquake in China, the 1990 earthquake in western Iran, and the 2005 earthquake in Pakistan, all occurred in Asia’s MSHA. The total death toll of those five earthquakes reached over 688 thousand people [50].

More importantly, the population living throughout Asia’s MSHA is expected to grow rapidly by 57.93 million people from 2015 to 2020 with a growth rate of 5.39%, which is 0.92% greater than the estimated population growth rate in the non-MSHA region (Table A5). In addition, the people living among the MSHA of South Asia, Central Asia, Southeast Asia, and West Asia are estimated to continue to increase rapidly with growth rates of 7.22%, 7.21%, 6.04% and 6.10%, respectively, all of which will be greater than the average population growth rate in the MSHA of Asia (Table A5). Correspondingly, the populations among the MSHA of those four regions will increase by 29.96 million, 3.52 million, 15.98 million, and 6.84 million people. At the national scale, the population growth rates among the MSHA of 23 countries will be greater than the average population growth rate of the population in Asia’s MSHA (Table A2 and Table A5). The population growth rates in the MSHA among those 23 countries will range from 5.61% to 16.13%, and the amount of population growth in the MSHA of those countries will reach 47.97 million people. Among the 23 countries, the growth rates of the population in the MSHA of Palestine, Iraq, and Syria will be particularly high at 16.13%, 15.32%, and 13.59%, respectively, which will be 2.5–3 times the average growth rate of the population in Asia’s MSHA (Table A2 and Table A5).

Meanwhile, the vulnerable population living throughout Asia’s MSHA is also expected to grow rapidly by 24.20 million people between 2015 and 2020 with a growth rate of 6.28%. Such a growth rate will be 0.43% greater than that of the vulnerable population in the non-MSHA region (Table A5). In addition, the vulnerable populations living in the MSHA of Central Asia, South Asia, and West Asia will all continue to increase rapidly with growth rates of 9.70%, 7.40%, and 7.18%, respectively, all of which will be greater than the average growth rate of the vulnerable population in Asia’s MSHA (Table A5). Correspondingly, the vulnerable populations in the MSHA of those three regions will increase by 1.62 million, 12.04 million, and 2.83 million people. At the national scale, the vulnerable population growth rates in the MSHA of 20 countries will be greater than the average population growth rate in Asia’s MSHA (Table A3 and Table A5). The growth rates of the vulnerable populations among the MSHA of those 20 countries will vary between 7.14% and 23.66%, and their total vulnerable population growth in the MSHA will reach 13.49 million people. Among those 20 countries, the growth rate of the vulnerable population living in the MSHA of Oman will be the highest with a value of 23.66%, which will be 3.77 times the average growth rate of the vulnerable population in Asia’s MSHA (Table A3 and Table A5).

Therefore, more attention should be paid to the changes of the population in Asia’s MSHA. First, the monitoring scheme for the population changes in Asia’s MSHA should be enhanced. Asian countries should further strengthen their construction of real-time monitoring platforms for the populations living throughout the MSHA. This is especially true for countries such as the United Arab Emirates, Oman, and Afghanistan with rapidly increasing populations living in the MSHA as well as for countries with rapidly increasing vulnerable MSHA populations (e.g., the United Arab Emirates, Lebanon, Jordan, and Iraq).

### 4.4. Future Perspectives

The current study exhibits several limitations. First, the PGA data in Asia possess some limitations [34]. The PGA data for Asia were generated by using the probabilistic seismic hazard assessment method, which may underestimate the seismic hazard [51]. Second, the input model parameters for the population distribution data in the WorldPop datasets for Asia were derived from the spatial population data of numerous countries with various spatial resolutions. Therefore, the accuracy of the WorldPop data may be limited to a certain extent [28]. Third, vulnerable population also included other groups, such as people with disabilities, those who have been displaced, and refugees [10]. Due to the limitation of acquiring the data for these groups of vulnerable population, we only considered children and elderly people as vulnerable populations in this study. Besides, we simply explore the correlation between several socioeconomic factors and the changes of the population in Asia’s MSHA, while the driving mechanisms still need to be explored.

To avoid the limitations of PGA data in future studies, we will further seek to employ PGA data based on loss scenarios with the maximum credible earthquake method [7]. In addition, we will consider the use of multisource remote sensing data and geospatial data or volunteered geographic information data to obtain high-accuracy spatial population data [52,53].

## 5. Conclusions

The population in Asia’s MSHA increased rapidly by 185.88 million between 2000 and 2015 with a growth rate of 20.93%. The growth rate of the population in the MSHA was 3.38% greater than that in the non-MSHA region. Over that same period, the vulnerable population in Asia’s MSHA also increased rapidly by 63.65 million with a growth rate of 19.73%. The growth rate of the vulnerable population in the MSHA was 19.93% greater than that in the non-MSHA region.

Urban population growth was a major factor associated with the increase in both the population and the vulnerable population in Asia’s MSHA. The Pearson correlation coefficients between the urban population growth and the population growth in Asia’s MSHA were 0.89 (*p* < 0.001). The GLM regression results further indicated that the urban population growth accounted for 65.87% of the variation in the population in the MSHA. Further analysis indicated that the correlation coefficient between the urban population growth and the vulnerable population growth in Asia’s MSHA was 0.61 (*p* < 0.001) and that urban population growth could account for 50.95% of the variations in the vulnerable population in the MSHA.

Historical data suggested the presence of high-frequency earthquakes throughout Asia’s MSHA, and the death toll collectively in Asia’s MSHA was enormous. More importantly, the population living throughout Asia’s MSHA, especially its vulnerable population, is expected to grow rapidly in the future. Therefore, more attention should be paid to changes in the population throughout Asia’s MSHA. The monitoring of population changes in Asia’s MSHA should be strengthened. Furthermore, earthquake risk prevention techniques should be fully considered in urban planning endeavors in the future, and the vulnerable population should be equipped with the knowledge of seismic disaster prevention and mitigation practices.

## Figures and Tables

**Table 1:** The area of MSHA.

Region	MSHA Area (10^4^ km^2^)	Regional Area (10^4^ km^2^)	Percentages of MSHA Area to the Regional Area (%)	Percentage of MSHA Area to the Total MSHA Area of Asia (%)
Asia	898.96	3115.84	28.85	
South Asia	304.44	668.22	45.56	33.87
East Asia	272.06	1153.54	23.58	30.26
Southeast Asia	137.65	445.82	30.88	15.31
West Asia	96.38	451.42	21.35	10.72
Central Asia	88.43	396.84	22.28	9.84

**Table 2:** Changes in the total population of Asia from 2000 to 2015.

Area	Region	2000	2015	2000 to 2015	Change Rate ^1^
		(Million)	(Million)	(Million)	(%)
MSHA	Asia	888.07	1073.95	185.88	20.93
	West Asia	85.16	112.22	27.07	31.78
	South Asia	326.03	414.75	88.73	27.21
	Central Asia	39.20	48.77	9.57	24.41
	Southeast Asia	215.62	264.73	49.11	22.78
	East Asia	222.06	233.47	11.41	5.15
non-MSHA	Asia	2797.24	3288.09	490.85	17.55
	West Asia	99.80	145.01	45.20	45.29
	South Asia	1125.91	1408.22	282.31	25.07
	Central Asia	15.89	18.54	2.65	16.71
	Southeast Asia	310.56	368.76	58.20	18.74
	East Asia	1245.08	1347.56	102.48	8.23

^1^ The change rate of the total population is calculated as (*P*_2015_ *− P*_2000_)/*P*_2000_ × 100%, whereas *P*_2015_ and *P*_2000_ refer to the total population in 2015 and 2000, respectively.

**Table 3:** Changes in the vulnerable population of Asia from 2000 to 2015.

Area	Region	2000	2015	2000 to 2015	Change Rate ^1^
		(Million)	(Million)	(Million)	(%)
MSHA	Asia	321.94	385.47	63.53	19.73
	Southeast Asia	75.04	92.47	17.43	23.23
	West Asia	32.45	39.38	6.93	21.33
	South Asia	134.40	162.77	28.37	21.11
	East Asia	64.13	74.16	10.03	15.65
	Central Asia	15.92	16.69	0.77	4.84
non-MSHA	Asia	1013.47	1011.40	−2.07	−0.20
	Southeast Asia	117.89	113.33	−4.56	−3.86
	West Asia	42.46	51.14	8.69	20.46
	South Asia	447.09	474.42	27.33	6.11
	East Asia	399.93	366.31	−33.62	−8.41
	Central Asia	6.10	6.19	0.09	1.48

^1^ The change rate of the vulnerable population is calculated as (*V*_2015_ *− V*_2000_)/*V*_2000_ × 100%, whereas *V*_2015_ and *V*_2000_ refer to the vulnerable population in 2015 and 2000, respectively.

**Table 4:** Selected factors associated with population changes in the MSHA from 2000 to 2015.

Method	Variable	Total Population in the MSHA		Vulnerable Population in the MSHA	
		*r*	*p*	*r*	*p*
Pearson’s Correlation	Population density	0.998	0.000	0.741	0.000
	Urban population	0.894	0.000	0.607	0.000
	GDP	0.127	0.419	0.037	0.603
	GDP per capita	0.379	0.183	0.308	0.111
	Urban land area	0.875	0.000	0.575	0.001
	Birth rate	−0.333	0.245	−0.205	0.278
	Infant mortality rate	0.399	0.157	0.201	0.287
Multiple GLM Regression	Variable	MS	SS, %	MS	SS, %
	Population density	1.396	32.82 *	0.15	26.19 *
	Urban population	2.802	65.87 *	0.30	50.95 *
	GDP	0.003	0.07	0.00	0.10
	Urban land area	0.016	0.37	0.01	1.18
	Birth rate	0.004	0.10	0.00	0.51
	Infant mortality rate	0.000	0.00	0.00	0.56
	Residuals	0.002	0.77	0.01	20.51

Note: Variables in the table represent the change rates of the factors from 2000 to 2015. The multiple GLM regression results passed the standard regression diagnostics. The variance inflation factors (VIFs) were tested as less than 8. All of the statistical analyses were conducted in R version 3.3.1 (http://www.R-project.org) (RStudio, Boston, The United States). * *p* < 0.05; MS: mean squares; SS: proportion of variances explained by the variables.

## Appendix A

**Table A1:** The areas of the MSHA in each country.

Region	Country	MSHA Area	Percentage of MSHA Area to the Area of Country	Percentage of MSHA Area in the Total MSHA Area of Asia
		(10^4^ km^2^)	(%)	(%)
West Asia	Turkey	59.48	76.14	6.62
	Iraq	8.45	19.33	0.94
	Azerbaijan	8.37	96.96	0.93
	Georgia	6.85	98.08	0.76
	Armenia	2.83	95.21	0.31
	Oman	2.76	8.97	0.31
	United Arab Emirates	2.11	29.75	0.23
	Syria	1.69	9.11	0.19
	Israel	1.02	46.40	0.11
	Lebanon	0.86	85.33	0.10
	Palestine	0.53	84.39	0.06
	Cyprus	0.50	55.79	0.06
	Jordan	0.50	5.59	0.06
	Saudi Arabia	0.43	0.22	0.05
South Asia	Iran	144.02	88.82	16.02
	India	53.26	16.89	5.92
	Pakistan	48.49	55.34	5.39
	Afghanistan	33.52	52.26	3.73
	Nepal	14.69	99.90	1.63
	Bangladesh	6.50	47.61	0.72
	Bhutan	3.96	99.68	0.44
Southeast Asia	Indonesia	71.17	37.68	7.92
	Myanmar	38.38	57.47	4.27
	Philippines	23.16	78.25	2.58
	Thailand	1.73	3.37	0.19
	Vietnam	1.48	4.52	0.16
	Laos	1.18	5.14	0.13
	Timor-Leste	0.55	37.20	0.06
Central Asia	Kyrgyzstan	19.25	96.61	2.14
	Uzbekistan	19.09	44.07	2.12
	Turkmenistan	18.49	37.78	2.06
	Kazakhstan	17.46	6.45	1.94
	Tajikistan	14.14	99.56	1.57
Eastern Asia	China	215.08	22.94	23.93
	Mongolia	29.41	18.78	3.27
	Japan	27.57	73.78	3.07

**Table A2:** Changes in the total population living among different MSHA from 2000 to 2020 in each country.

Region	Country	Total Population in the MSHA (Million)				Change Rate of the Total Population in the MSHA	
		2000	2015	2000–2015	2015–2020	2000–2015	2015–2020
West Asia	United Arab Emirates	2.03	6.68	4.65	0.68	228.41%	10.13%
	Oman	1.40	2.76	1.36	0.25	97.24%	9.18%
	Lebanon	2.46	4.43	1.97	0.03	80.18%	0.70%
	Jordan	1.01	1.62	0.61	0.13	59.82%	8.13%
	Iraq	5.53	8.54	3.00	1.31	54.25%	15.32%
	Saudi Arabia	0.03	0.04	0.01	0.00	46.64%	9.59%
	Palestine	1.77	2.51	0.75	0.41	42.39%	16.13%
	Israel	3.43	4.66	1.24	0.35	36.15%	7.43%
	Turkey	47.46	59.49	12.03	2.65	25.34%	4.46%
	Cyprus	0.65	0.80	0.16	0.03	24.01%	4.05%
	Azerbaijan	8.11	9.75	1.64	0.49	20.27%	4.98%
	Syria	3.46	3.91	0.45	0.53	13.06%	13.59%
	Armenia	3.08	3.02	−0.06	0.01	−2.02%	0.41%
	Georgia	4.74	4.00	−0.74	−0.03	−15.62%	−0.75%
South Asia	Afghanistan	14.71	24.29	9.58	2.91	65.16%	11.98%
	Bhutan	0.56	0.77	0.21	0.05	38.36%	5.83%
	Pakistan	70.13	95.85	25.72	9.92	36.68%	10.35%
	India	103.75	129.03	25.28	7.73	24.36%	5.99%
	Bangladesh	54.87	67.20	12.33	4.05	22.47%	6.03%
	Nepal	23.75	28.51	4.76	1.68	20.06%	5.88%
	Iran	58.26	69.09	10.83	3.63	18.60%	5.25%
Southeast Asia	Timor-Leste	0.20	0.27	0.08	0.03	39.70%	11.07%
	Philippines	57.95	74.97	17.02	5.66	29.37%	7.55%
	Laos	0.31	0.39	0.08	0.04	27.05%	8.93%
	Indonesia	132.41	161.12	28.71	9.04	21.68%	5.61%
	Vietnam	0.66	0.76	0.11	0.04	15.99%	5.34%
	Myanmar	23.56	26.63	3.07	1.17	13.02%	4.40%
	Thailand	0.54	0.58	0.05	0.00	8.75%	0.62%
Central Asia	Tajikistan	6.18	8.48	2.30	0.95	37.25%	11.18%
	Turkmenistan	1.59	1.97	0.39	0.15	24.30%	7.62%
	Uzbekistan	21.24	26.18	4.94	1.68	23.26%	6.40%
	Kyrgyzstan	4.95	5.94	0.99	0.45	20.00%	7.58%
	Kazakhstan	5.25	6.20	0.95	0.33	18.14%	5.35%
East Asia	Mongolia	0.28	0.35	0.07	0.03	23.44%	7.42%
	China	128.45	139.19	10.74	2.70	8.36%	1.94%
	Japan	93.34	93.94	0.60	−1.10	0.64%	−1.17%

^1^ The change rate of the total population from 2000 to 2015 is calculated as (*P*_2015_ *− P*_2000_)/*P*_2000_ × 100%, where *P*_2015_ and *P*_2000_ refer to the total population in 2015 and 2000, respectively. ^2^ The change rate of the total population from 2015 to 2020 is calculated as (*P*_2020_ *− P*_2015_)/*P*_2015_ × 100%, where *P*_2020_ and *P*_2015_ refer to the total population in 2020 and 2015, respectively.

**Table A3:** Changes in the vulnerable population living among different MSHA from 2000 to 2020 in each country.

Region	Country	Vulnerable Population in the MSHA (Million)				Change Rate of the Vulnerable Population in the MSHA	
		2000	2015	2000–2015	2015–2020	2000–2015	2015–2020
West Asia	United Arab Emirates	0.54	1.01	0.47	0.17	86.33%	17.34%
	Lebanon	0.88	1.42	0.54	−0.05	61.94%	−3.23%
	Iraq	2.58	3.98	1.40	0.61	54.28%	15.32%
	Jordan	0.43	0.64	0.21	0.02	47.91%	3.72%
	Israel	1.31	1.82	0.52	0.18	39.49%	10.01%
	Turkey	17.83	22.10	4.28	0.90	23.98%	4.08%
	Palestine	0.88	1.09	0.21	0.14	23.29%	13.12%
	Oman	0.55	0.64	0.08	0.15	15.10%	23.66%
	Saudi Arabia	0.01	0.01	0.00	0.00	14.68%	7.14%
	Cyprus	0.21	0.24	0.02	0.02	11.73%	7.85%
	Syria	1.53	1.61	0.08	0.04	5.37%	2.43%
	Azerbaijan	2.97	2.69	−0.28	0.47	−9.54%	17.62%
	Armenia	1.11	0.88	−0.23	0.08	−20.37%	9.14%
	Georgia	1.63	1.25	−0.38	0.08	−23.06%	6.06%
South Asia	Afghanistan	7.27	12.00	4.74	1.44	65.16%	11.98%
	Pakistan	33.06	45.18	12.12	4.68	36.68%	10.35%
	India	37.56	46.70	9.14	2.81	24.35%	6.01%
	Bangladesh	22.82	27.95	5.13	1.68	22.48%	6.03%
	Nepal	10.64	10.90	0.26	−0.26	2.40%	−2.34%
	Bhutan	0.25	0.25	0.00	0.00	−1.04%	1.06%
	Iran	22.80	19.79	−3.02	1.69	−13.22%	8.55%
Southeast Asia	Timor-Leste	0.10	0.14	0.04	0.02	39.70%	11.07%
	Philippines	23.59	30.52	6.93	2.30	29.38%	7.55%
	Laos	0.13	0.16	0.03	0.01	27.12%	8.87%
	Indonesia	43.45	52.87	9.42	2.97	21.68%	5.62%
	Vietnam	0.26	0.30	0.04	0.02	16.07%	5.28%
	Myanmar	7.34	8.30	0.96	0.36	13.02%	4.40%
	Thailand	0.17	0.19	0.02	0.00	8.75%	0.62%
Central Asia	Turkmenistan	0.53	0.66	0.13	0.05	24.18%	7.72%
	Kazakhstan	1.73	2.05	0.32	0.11	18.19%	5.31%
	Tajikistan	2.87	3.21	0.34	0.45	11.84%	14.02%
	Kyrgyzstan	2.00	2.12	0.12	0.29	5.82%	13.88%
	Uzbekistan	8.79	8.66	−0.13	0.72	−1.48%	8.26%
East Asia	Japan	29.69	36.82	7.14	1.29	24.04%	3.51%
	Mongolia	0.12	0.15	0.03	0.01	23.43%	7.42%
	China	34.32	37.20	2.87	0.72	8.37%	1.94%

^1^ The change rate of the vulnerable population from 2000 to 2015 is calculated as (*V*_2015_ *− V*_2000_)/*V*_2000_ × 100%, where *V*_2015_ and *V*_2000_ refer to the vulnerable population in 2015 and 2000, respectively. ^2^ The change rate of the vulnerable population from 2015 to 2020 is calculated as (*V*_2020_ *− V*_2015_)/*V*_2015_ × 100%, where *V*_2020_ and *V*_2015_ refer to the vulnerable population in 2020 and 2015, respectively.

**Table A4:** Earthquake events in the MSHA between 1900 and 2009.

Earthquake Intensity	Total Earthquakes in Asia (Number)	Total Earthquakes in the MSHA (Number)	Percentage of the Total Earthquakes in the MSHA (%)
Total	1824	1500	82.24
5.5–6.0	916	735	80.24
6.0–6.5	498	417	83.73
6.5–7.0	253	216	85.38
7.0 above	157	132	84.08

Note: These data were acquired from the Global Instrumental Earthquake Catalogue released by the International Seismological Centre—Global Earthquake Model [47].

**Table A5:** Expected changes in the total population and in the vulnerable population of Asia from 2015 to 2020.

Area	Region	Total Population Change	Change Rate of the Total Population *	Vulnerable Population Change	Change Rate of the Vulnerable Population **
		(Million)	(%)	(Million)	(%)
MSHA	Asia	57.93	5.39	24.20	6.28
	South Asia	29.96	7.22	12.04	7.40
	Central Asia	3.52	7.21	1.62	9.70
	West Asia	6.84	6.10	2.83	7.18
	Southeast Asia	15.98	6.04	5.68	6.15
	East Asia	1.63	0.70	2.03	2.73
non-MSHA region	Asia	146.94	4.47	59.21	5.85
	South Asia	87.25	6.20	8.45	1.78
	Central Asia	1.07	5.78	0.85	13.75
	West Asia	15.04	10.37	3.84	7.50
	Southeast Asia	18.17	4.93	3.91	3.45
	East Asia	25.41	1.89	42.16	11.51

* Please refer to Table 2 for the calculation of the change rate of the total population. ** Please refer to Table 3 for the calculation of the change rate of the vulnerable population.
